# Hand function following accidental automatic animal syringe injector injuries

**DOI:** 10.1038/s41598-022-25641-0

**Published:** 2022-12-06

**Authors:** Guy Rubin, Guy Feldman, Shtawe Shtawe, Nimrod Rozen

**Affiliations:** 1 Orthopedic Department, Emek Medical Center, Afula, Israel; 2 Faculty of Medicine, Technion, Haifa, Israel

**Keywords:** Health care, Infectious diseases, Trauma

## Abstract

Accidental self-injection injury is a common occurrence among veterinary and farm workers handling automatic syringe injectors. Most of the time, these injuries are asymptomatic or cause self-resolving mild symptoms, but these injuries may lead to significant morbidity. The aim of the study was to evaluate hand function after inadvertent injection of a poultry influenza or cholera vaccine in patients admitted to our department with infection. We retrospectively gathered data from admission to last follow-up. Functional assessment and physical exam of the hand were done at each stage by either an orthopedic resident or a fellowship-trained hand surgeon. The exam included evaluation of sensation using monofilament, joint range of motion using a goniometer, and a Quick DASH questionnaire. The study included 21 patients, all men, with a mean age of 33.4 years (range 23–44). Of the 21 patients only eight had attended all follow-ups. All patients had injury to the non-dominant hand. Seventeen of 21 of the cases had finger injuries, out of which 11 involved the thumb. The mean hospitalization time was 3.75 days (1–10). Of the 21 patients, seven underwent surgery to drain a collection during hospitalization. Seven out of eight patients had lowest disability scores on Quick Dash questionnaire. Three out of eight patients lost superficial sensation at the tip of the finger. The largest loss of range of motion was found in the distal interphalangeal joint in the finger or interphalangeal joint in the thumb, especially following surgical drainage. Of the eight patients presenting for follow-up, most had returned to the same job. Hand function was normal, as expressed in a DASH questionnaire. Sensory examination demonstrated that the sensation was almost unaffected over the injured finger. Range of motion of the joint closest to the injection site was usually the most impaired. Patients who underwent surgical drainage had a reduced range of motion.

## Introduction

**Figure 1:**
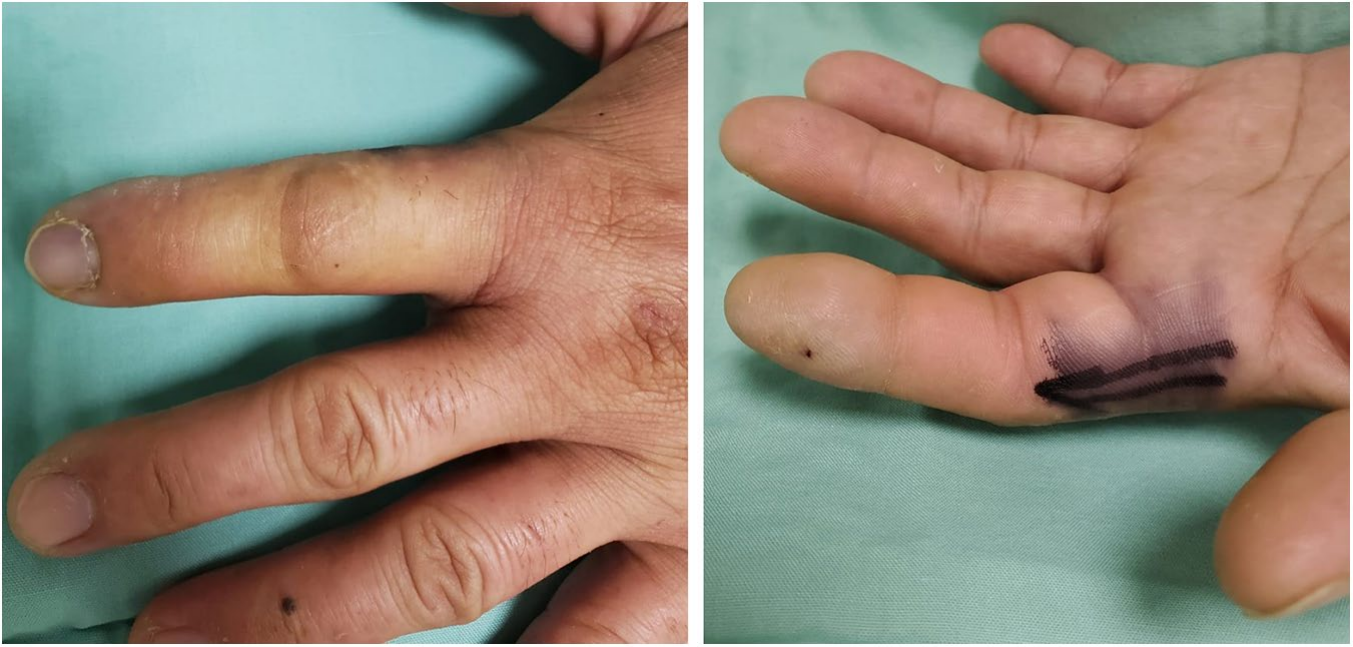
(**a**,**b**) Index injection injury with collection prior to drainage following automatic syringe injector injury.

Accidental self-injection injury is a common occurrence among veterinary and farm workers handling automatic syringe injectors (ASI), used in mass vaccinations of animals. In these populations, the lifetime occurrence of accidental injection during vaccination of an animal ranges from 64 to 93%. Incidence is probably much higher, as these injuries are usually not reported^1–6^. Fortunately, most of these injuries are asymptomatic or cause only mild, self-limited symptoms, but a portion of these injuries will lead to significant morbidity, including local and systemic consequences, such as infection^7–9^, soft tissue necrosis^10^, and the need for extensive surgical debridement or amputation of a digit^11^ (Fig. 1a,b).

Similar to high-pressure injection injuries of the digit, seen in workers handling diesel injection apparatus or high-pressure paint sprayers, the severity of injuries is related to several variables including location, needle trajectory, secondary bacterial infection, and the type of inoculum^12^. ASI injuries usually involves the non-dominant hand^13^, Inexperienced male employees who have worked less than 6 months are at a higher risk of injury^14^.

Kaufman et al.^15^ dissected cadaver hands following a simulated pressure injection injury and mapped the pattern of anatomical spread of liquid material at various anatomical regions of the hand. He found that the injected liquid substance will spread in tissues until it encounters an impenetrable structure. He also stated that substances will rarely spread to the side of the injection trajectory. Tissues that resist penetration and spread of the liquid include bone, tendon, and flexor sheath. Hand anatomy is susceptible to these injuries, as it displays a lack of anatomical buffer zones, such as fat, and an abundance of terminal arteries and nerves, which can lead to increased morbidity in case of these injuries when compared to other areas of the body. ASI injuries are different than high-pressure injection injuries found in diesel and paint industries, which have higher finger amputations rates. Hogan and Ruland^16^ studied the outcome of high-pressure injuries and showed an overall amputation rate of 47% after finger injections, compared to 15% for the thumb and 25% for the dorsum of the hand.

The choice of antibiotic coverage is made empirically to cover common skin contaminants (Staphylococcus and Streptococcus), as well as organisms that may be present in the agricultural environment, including anaerobic bacteria and bacteria from the gram-negative family. Colonization of antibiotic-resistant organisms (methicillin resistant Staphylococcus aureus) is common in agricultural industry workers and thus should be considered when choosing antibiotic regimens in this population^17^.

We did not find any evidence of functional outcome documentation of ASI injuries in humans, but, in general, increasing the volume of injection in the finger compartment and the fact that the same needle is used repeatedly, and becomes contaminated with bacteria from the environment can lead to an increased risk of infection, necrosis and subsequent amputation^14,18^.

This clinical question of this study is whether the workers injured their hand from poultry influenza or cholera vaccine and admitted our department had functional deficit?

## Patients and methods

This is a retrospective descriptive study done between the years 2013 and 2021. This study was approved by the Emek medical center review bord (EMC 24-21), all methods were performed in accordance with the relevant guidelines and regulations. Patients admitted following ASI injury that occurred during poultry vaccination, containing either influenza or cholera vaccine were included. Our hypothesis was that admitted patients will have functional deficit due to this injury.

Data was retrospectively collected using computerized files, with information from admission to last follow-up collected. Functional assessment and physical examination of the hand were performed in each stage by either an orthopedic resident or a fellowship-trained hand surgeon. The examinations completed for available patients included evaluations of sensation using monofilament, joint range of motion using a goniometer, and the Quick DASH questionnaire (The QuickDASH tool uses a 5-point Likert scale from which the patient can select an appropriate number corresponding to his/her severity/function level)^19^.

### Ethical approval

Ethical approval to report these cases was obtained from the Emek medical center review bord (APPROVAL NUMBER/0120-21-EMC).

Written informed consent was obtained from the patient(s) for their anonymized information to be published in this article.

The authors have read the Journal's position on issues involved in ethical publication.

## Results

The study included 21 patients, all men, with a mean age of 33.4 years (range 23–44). Of the 21 patients, only eight had attended all follow-ups. Table 1 displays patient's demographics. All patients had injury to the non-dominant hand with no documentation of an old injury. Both volar and dorsal side of the hand were found. Seventeen of 21 of the cases had finger injuries, of which 11 involved the thumb.

**Table 1:** Demographics and injury characteristics.

Number	Age	Side of injury	Dominant hand	injured area	Injured digit	Digit zone of injury
1	26	Left	Right	Volar	2	Pulp
2	36	Left	Right	Dorsal	1	Proximal phalange
3	30	Left	Right	Volar	1	Pulp
4	34	Left	Right	Volar	2	Proximal phalange
5	31	Left	Right	Dorsal	1	Distal phalange
6	34	Left	Right	Dorsal	1	Metacarpal
7	24	Right	Left	Volar	1	Pulp
8	34	Left	Right	Volar	2	Pulp
9	41	Right	Left	Volar	2	Pulp
10	44	Left	Right	Dorsal	WEB-1	WEB-1
11	43	Left	Right	Dorsal	Metacarpal	Metacarpal
12	32	Left	Right	Dorsal	Forearm	Forearm
13	30	Right	Left	Dorsal	5	Proximal phalange
14	28	Left	Right	Dorsal	1	Metacarpal
15	40	Left	Right	Volar	1	Proximal phalange
16	40	Left	Right	Dorsal	1	Metacarpal
17	28	Left	Right	Dorsal	1	Metacarpal
18	29	Left thigh	Right	–	Thigh	
19	41	Left	Right	Volar	1	Pulp
20	23	Left	Right	Volar	1	Pulp
21	26	Left	Right	Volar	2	Pulp

The mean hospitalization time was 3.75 days (1–10). Of the 21 patients, seven underwent surgery to drain a collection during hospitalization (only one patient underwent two repeated procedures).

Of the seven patients who underwent surgery, only three had positive cultures. The latter is due to antibiotic treatment that preceded the surgical drainage. Cephazolin or Augmentin were used for empiric antibiotic treatment. Antibiotics were adjusted after obtaining susceptibility results (Table 2).

**Table 2:** Results.

Number	Surgery	Culture	Antibiotics	Length of hospitalization in days	Time elapsed from last follow-up in months	Return to work	Quick dash	Monofil test	Range of motion loss MCP in degrees	Range of motion loss PIP in degrees	Range of motion loss DIP in degrees
1	0	NA	Cefamezin	2	14	Yes	0	Intact	NA	NA	Extension 5 Flexion 10
2	0	NA	Cefamezin	4	14	Yes	4.5	Intact	NA	Not relevant	Extension 5 Flexion 5
3	1	Pseudomonas	Fortum + cipro	10	29	Yes	0	Intact	NA	Not relevant	Extension 0 Flexion 20
4	1	No growth	Cefamezin	3	18	Yes	0	2.83	NA	NA	Extension 0 Flexion 45
5	1	Staph. Epidermidis	Cefamezin	4	3	NA	2.3	2.84	NA	Not relevant	Extension 0 Flexion 5
6	0		Cefamezin and augmentin	8	4	Yes	36.4	3.61	5 Flexion	Not relevant	Extension 0 Flexion 20
7	1	No growth	Cefamezin	3	19	NA	0	3.61	NA	Not relevant	Extension 0 Flexion 5
8	2	No growth	Cefamezin	5	3	Yes	13.6	3.61	5 Flexion	5 Flexion	Extension 0 Flexion 45
9	1	No growth	Augmentin	6	2	NA	NA	NA	NA	NA	NA
10	0		Cefamezin augmentin	3	NA	NA	NA	NA	NA	NA	NA
11	0		Cefamezin	3	NA	NA	NA	NA	NA	NA	NA
12	0		Cefamezin	3	NA	NA	NA	NA	NA	NA	NA
13	0		Cefamezin	3	NA	NA	NA	NA	NA	NA	NA
14	0		Cefamezin	2	NA	NA	NA	NA	NA	NA	NA
15	0		Augmentin	5	NA	NA	NA	NA	NA	NA	NA
16	0		Augmentin	4	NA	NA	NA	NA	NA	NA	NA
17	0		Augmentin	1	NA	NA	NA	NA	NA	NA	NA
18	1	Staph. Aureus	Cefamezin	3	NA	NA	NA	NA	NA	NA	NA
19	0		Cefamezin	2	NA	NA	NA	NA	NA	NA	NA
20	0		Cefamezin	1	NA	NA	NA	NA	NA	NA	NA
21	1	No growth	Cefamezin	2	NA	NA	NA	NA	NA	NA	NA

The average follow-up time was 11.7 months (3–29). Six of the eight patients returned to work, while two chose not to return to the same job for reasons unrelated to the injury. Seven out of eight patients achieved the lowest disability score on Quick Dash questionnaire. Only one patient presented a score of 36.4, indicating a minor disability of the hand. Three out of eight patients lost superficial sensation at the tip of the finger. Range of motion was measured with a goniometer for all injured finger joints. The metacarpophalangeal joint did not lose range of motion except for five degrees of flexion in two patients, the proximal interphalangeal joint lost 5 degrees of flexion in one patient, and the distal interphalangeal joint in the finger and the interphalangeal joint in the thumb both lost the most range of motion, especially following surgical drainage (between 5 to 45 degrees of flexion).

## Discussion

This study demonstrated, for the first time, the clinical outcomes in patients hospitalized after accidental injection injury to the fingers resulted from farm use of automatic syringe injectors. Previous studies have estimated that accidental injection injury is extremely common^1–6^, Leggat et al.^3^ reported a survey of 664 veterinarians with around three quarters (75.3%) reported suffering at least one NSI in the previous 12 months, while 58.9% reported suffering from at least one contaminated NSI during the previous 12 months. Weese et al.^5^ reported that 74% had experienced a needlestick injury during the preceding year. Nevertheless, according to current data, only a minority of cases develop local infections requiring antibiotic treatment, and of these cases, only a few require hospitalization for parenteral antibiotic treatment or surgery.

Vaccines for animals are rarely performed with single-use sterile needles. When mass vaccination of animals is done using an ASI, the same needle is used multiple times, compromising its sterility. Jenissan et al. documented a series of nine cases of accidental self-injection during animal vaccination. Five out of the patients developed infections requiring broad spectrum antibiotic treatment. In three individuals, empirical antibiotic treatment was needed due to infection with cellulitis, lymphangitis, and negative cultures. Two patients with infection had positive cultures for Streptococcus and Streptomyces, respectively^13^. Of our 21 patients, seven underwent surgery to drain a collection during hospitalization, and only three had positive cultures.

This study summarizes our experience with 21 patients over more than 8 years. The limitation of this study is its retrospective nature and the small number of patients that were available for long follow-up. This injury is common, the lifetime occurrence of accidental injection during vaccination of an animal ranges from 64 to 93%^1–6^, but the number of workers who have complication such an infection^7–9^ is very small and the evaluability of them in long follow-up is even smaller. Of the eight patients who did present for follow-up, most had returned to the same job. Hand function was normal as expressed in a DASH questionnaire. Sensory examination demonstrated that the sensation was almost unaffected over the injured finger. Range of motion of the joint closest to the injection site was usually most impaired. Patients who had drainage surgery had reduced range of motion.

## Data Availability

The datasets used and/or analyzed during the current study available from the corresponding author on reasonable request.
